# An alternative approach of TUNEL assay to specifically characterize DNA fragmentation in cell model systems

**DOI:** 10.1007/s00418-024-02306-9

**Published:** 2024-06-28

**Authors:** Flores Naselli, Paola Sofia Cardinale, Sara Volpes, Chiara Martino, Ilenia Cruciata, Rossella Valenti, Claudio Luparello, Fabio Caradonna, Roberto Chiarelli

**Affiliations:** 1 Department of Biological, Chemical and Pharmaceutical Sciences and Technologies (STEBICEF), University of Palermo, Viale Delle Scienze Building 16, 90128 Palermo, Italy; 2 NBFC, National Biodiversity Future Center, 90133 Palermo, Italy

**Keywords:** DNA damage, DNA fragmentation, Pyknosis, Genomic instability, Single chromosomes fragmentation

## Abstract

DNA damage is one of the most important effects induced by chemical agents. We report a comparative analysis of DNA fragmentation on three different cell lines using terminal deoxynucleotidyl transferase dUTP nick end labeling (TUNEL) assay, generally applied to detect apoptosis. Our approach combines cytogenetic techniques and investigation in detached cellular structures, recovered from the culture medium with the aim to compare the DNA fragmentation of three different cell line even beyond the cells adherent to substrate. Consequently, we detect any fragmentation points on single chromosomes, whole nuclei and other cellular structures. Cells were exposed to resveratrol (RSV) and doxorubicin (Doxo), in single and combined treatments. Control and treated astrocytes showed DNA damage in condensed nuclei and detached structures. Caco-2 cells showed fragmented DNA only after Doxo-treatment, while controls showed fragmented chromosomes, indicating DNA damage in replicating cells. MDA-MB-231 cells showed nuclear condensation and DNA fragmentation above all after RSV-treatment and related to detached structures. This model proved to perform a grading of genomic instability (GI). Astrocytes show a hybrid level of GI. Caco-2 cells showed fragmented metaphase chromosomes, proving that the DNA damage was transmitted to the daughter cells probably due to an absence of DNA repair mechanisms. Instead, MDA–MB-231 cells showed few or no fragmented metaphase, suggesting a probable activation of DNA repair mechanisms. By applying this alternative approach of TUNEL test, we obtained data that can more specifically characterize DNA fragmentation for a suitable application in various fields.

## Introduction

DNA fragmentation refers to the process of DNA strand breakage, resulting in the generation of smaller DNA fragments (Yoshida et al. 2006). This phenomenon has garnered significant attention in scientific research due to its relevance in various biological contexts (González-Marín et al. 2012). The distinctive nature of DNA fragmentation serves as a crucial cellular signature, providing valuable insights into fundamental cellular processes and pathological conditions (Kroemer et al. 2009; Kitazumi and Tsukahara 2011).

Understanding the mechanisms and consequences of DNA fragmentation is of paramount importance as it has implications in various biological phenomena, such as aging, cancer, response to therapeutic agents (Bouwman and Jonkers 2012; Chatterjee and Walker 2017; Ou and Schumacher 2018; Razak et al. 2019; Carusillo and Mussolino 2020), and embryonic development (Chiarelli et al. 2014, 2019, 2021, 2022c; Martino et al. 2021).

Interestingly, DNA fragmentation represents a relevant marker of the damage induced by physical or chemical stresses (Chiarelli and Roccheri 2012), especially when the cytoprotection mechanisms are not enough to deal with the stress (Agnello et al. 2016; Chiarelli et al. 2016, 2022a, b; Martino et al. 2021).

DNA fragmentation can also occur in response to various stressors, including genotoxic insults, oxidative damage, or DNA replication errors. Unrepaired or improperly repaired DNA breaks may result in aberrant DNA fragmentation patterns, leading to genomic instability and the potential for the development of genetic disorders or cancer (Jackson and Bartek 2009; Kroemer et al. 2009; Bouwman and Jonkers 2012; Caradonna 2015; Chatterjee and Walker 2017; Luparello et al. 2021).

In physiological conditions, DNA fragmentation occurs as a controlled and regulated process during specific stages of the cell cycle, or programmed cell death (Xu et al. 2019). During apoptosis, cells undergo a series of well-defined molecular events, culminating in the fragmentation of genomic DNA into smaller fragments of distinct sizes (Zhang and Xu 2000). For these reasons, DNA fragmentation is now considered a distinctive trait and a marker of irreversible cell death (Moore et al. 2021).

This characteristic DNA fragmentation pattern can be detected and analyzed using techniques such as DNA laddering gel electrophoresis, comet assay, terminal deoxynucleotidyl transferase dUTP nick end labeling (TUNEL) assay (Majtnerová and Roušar 2018), or next-generation sequencing (NGS) approaches (van Dijk et al. 2018). DNA laddering assay is not quantitative (Tsukada et al. 2001). Comet assay is limited to in vitro (cultured cells) only, and its quantification is labor-intensive (Kim et al. 2002). TUNEL is the most sensitive, accurate, and quantitative test (Moore et al. 2021), applicable to different biological samples.

In this study, we aim to conduct a comparative analysis of DNA fragmentation in different cell lines using the TUNEL assay on metaphase chromosomes samples, nuclei and cellular structures recovered from cultures media.

The TUNEL assay, initially designed as a standard histochemical method to detect DNA fragmentation resulting from apoptosis (Gavrieli et al. 1992), is the conventional approach followed in literature for analyzing cultured cells, cells in suspension and tissues.

Here, we propose an alternative approach that introduces two new and important variants: (1) cytogenetic techniques for selecting only those cells undergoing mitosis and (2) extension of the analysis of DNA fragmentation signals beyond the cells adherent to substrate. In fact, it has been recently described that the conditioned medium is a reservoir of useful information which until now was excluded from the various analyses because it was judged to be waste (Rosochowicz et al. 2023).

To achieve our objectives, clone-7 astrocytes and Caco-2 and MDA-MB-231 cells were subjected to single and combined treatments with resveratrol (RSV) and doxorubicin (Doxo) to modulate the level of DNA damage (Lin et al. 2014; Liu et al. 2014; Bong et al. 2020; Gomes et al. 2020; Jadid et al. 2021; Maszczyk et al. 2022; Volpes et al. 2023).

Investigating DNA fragmentation patterns at different levels and their underlying mechanisms contributes to the understanding of fundamental cellular processes, disease pathogenesis, and the development of potential diagnostic and therapeutic strategies. In particular, this study, by using an alternative approach of TUNEL assay, seeks to shed some light on the contribution of DNA fragmentation in different cell lines, elucidating the global profile of DNA damage.

## Materials and methods

### Cell lines and treatments

Clone-7 astrocytes cells were obtained by limited dilution technique starting from primary astrocytes, isolated as previously described (Caradonna et al. 2020).

Cells were cultured with Dulbecco’s modified Eagle’s medium (DMEM) high glucose (Sigma-Aldrich, D6429, USA) and F-12 K (Corning, 10–025-CV, USA) in a 2:1 ratio, enriched with 10% fetal bovine serum, 2 mM l-glutamine (Sigma-Aldrich, G7513, USA), and antibiotics penicillin/streptomycin 1% (Sigma-Aldrich, P4333, USA), until subconfluent, changing the culture medium approximately every 2 days and keeping cells at 37 °C in a controlled atmosphere (5% CO_2_).

Caco-2 and MDA-MB-231 cell lines were obtained from the American Type Culture Collection (Rockville, Md., USA) and cultured in DMEM (Sigma-Aldrich, D6429, USA) supplemented with 10% fetal bovine serum, 2 mM l-glutamine (Sigma, Life Science, G7513, U.K), and antibiotics penicillin/streptomycin 1% (Sigma-Aldrich, P4333, USA), and were maintained at 37 °C in 5% CO_2_ and 95% humidity. Caco-2 and MDA-MB-231 cell lines were cultured as previously described (Naselli et al. 2015; Luparello et al. 2019).

To identify potential modulatory effects on DNA fragmentation, clone-7 astrocytes and Caco-2 and MDA-MB-231 cells were treated with doxorubicin and resveratrol, solubilized in DMSO verifying that the vehicle volume was always less than 0.01% of the cell medium to avoid self-effect on the biological and epigenetic/molecular endpoints (Galvao et al. 2014).

The selected concentration of resveratrol for each cell line was 50 μM, chosen as it is under the IC_50_ reported in the literature (Lin et al. 2014; Liu et al. 2014; Gomes et al. 2019, 2020; Jadid et al. 2021; Volpes et al. 2023). The treatment extended for 48 h.

Clone-7 astrocytes and Caco-2 cells were also treated with 1 μM doxorubicin for 48 h (Jadid et al. 2021; Maszczyk et al. 2022), unlike MDA-MB-231 cells that were treated for 48 h with 0.6 μM doxorubicin chosen as it is under the IC_50_ reported in the literature (Sadeghi-Aliabadi et al. 2012).

Combined treatments with both selected molecules were carried out at the same concentrations and times used in the single treatments.

### Preparation of metaphase chromosomes

To perform the cytogenetic analysis, the cells were cultured in T75 cm^2^ flasks until reaching 70–80% confluence, thus ensuring the maintenance of the exponential phase of growth without reaching the stationary phase. Metaphases were obtained by adding colcemid (KaryoMAX^™^ Colcemid^™^ solution in HBSS, Gibco, 15,210–040, USA) at a final concentration of 0.1 μg/ml, 3 h before the trypsinization. The cultures were then processed according to the conventional air-drying protocol, as previously described (Sirchia and Luparello 2007; Mannino et al. 2019; Caradonna et al. 2020).

### Preparation of detached cell population

After cell culture, the medium was recovered and the pellet was washed two times in phosphate-buffered saline (PBS). The pellet was resuspended and fixed in 3.7% paraformaldehyde for 60 min, harvested by centrifugation at 200*g* for 7 min and resuspended in PBS. The slides were prepared by cytospin centrifugation at 60 *g* for 5 min on polylysine-coated glass slides. The glass permeabilization was performed at 4 °C in 0.1% Triton X-100 plus 0.1% sodium citrate in PBS. Therefore, after these steps we proceeded with the application of the TUNEL assay.

### TUNEL assay

The TUNEL assay (Promega, G3250, USA) was used to identify DNA fragmentation as described with some modifications for metaphase chromosomes and the detached cell population. The slide areas containing the biological material were delimited using a PAP Pen Liquid Blocker to create a visible hydrophobic barrier.

The slides were placed in a humid chamber and 50 μl of equilibration buffer (200 mM potassium cacodylate, pH 6.6 at 25 °C; 25 mM Tris–HCl, pH 6.6 at 25 °C; 0.2 mM DTT 0.25 mg/ml BSA; 2.5 mM cobalt chloride) was added in each area for 10 min. After, a mix containing: 1 μl of recombinant terminal deoxynucleotidyl transferase ( (rTdT) independent template DNA polymerase; 5 μl of nucleotide mix (fluorescein-12-dUTP) and 45 μl of equilibration buffer was added.

The reaction was carried on for 75 min at 37 °C and then stopped by immersing the slides in 2× salinesodium citrate (SSC) for 15 min. After two washes in PBS, propidium iodide (1:1000 dilution) was added for 10 min. After two washes in PBS, 10 μl of 1,4-diazabicyclo (2-2-2) octane, Tris–HCl 1 M pH 8, glycerol (DABCO) was added on each area. Finally, a coverslip was positioned and the fragmented labeled DNA in nuclei or chromosomes or in apoptotic body-like structures was observed by fluorescence microscopy (Olympus BX50, Japan) under a 60× objective.

Several microscope fields were acquired to obtain a significant number of observed cells. In addition, negative and positive controls were analyzed: negative controls were incubated with a buffer containing the fluorescent nucleotide mix (fluorescein 12-dUTP) without the TdT enzyme; positive controls were pretreated with DNAse (10 mg/ ml) for 10 min before the TdT assay, to fragment the total DNA.

Image acquisition was performed by measuring the intensity of autofluorescence in the negative control; this value was considered as the threshold level for the analysis of other samples. Any fluorescence intensity above the negative control’s threshold was therefore considered a positive TdT signal.

The amount of fragmented DNA and nuclei area were analyzed and quantified by ImageJ 1.46r image analyzing software after acquisition of different images using a digital camera (Nikon Sight DS-U1, Japan). This analysis was not applied to the images of the detached cells as there were no defined nuclei present but cellular structures in suspension containing DNA, fragmented or not.

The relative nuclei area was reported by setting the control equal to 1 and comparing the treated samples to this value. In this way, the values relating to the variation of the area of the nuclei in the treated cells indicate a variation of this parameter compared with the nuclear area of the control cells.

### Statistical analysis

Data of nuclei fragmentation and DNA fragmentation from detached cellular structures were presented as the mean of three independent experiments ± standard deviation (SD). The data were analyzed by one-way analysis of variance (ANOVA), using the Levene’s test to check homogeneity of variance and the Tukey’s HSD test as post hoc test analysis of significant differences. The area of fragmented and unfragmented nuclei in clone-7 astrocytes, and Caco-2 and MDA-MB-231 cells was presented as the mean of three independent experiments ± SD and comparisons between mean values for each group were carried using a *t*-test. The analyses were performed using the Statistica 13.2 software (StatSoft, Tulsa, OK, USA), and the level of significance was set to *p* < 0.05.

## Results

To evaluate a possible DNA damage in control and treated cells, analyses on several levels of DNA organization were carried out in all the considered cell lines: nuclei, metaphases and cellular structures recovered from the culture medium.

### Analysis of DNA fragmentation in clone-7 astrocytes

**Fig. 1:**
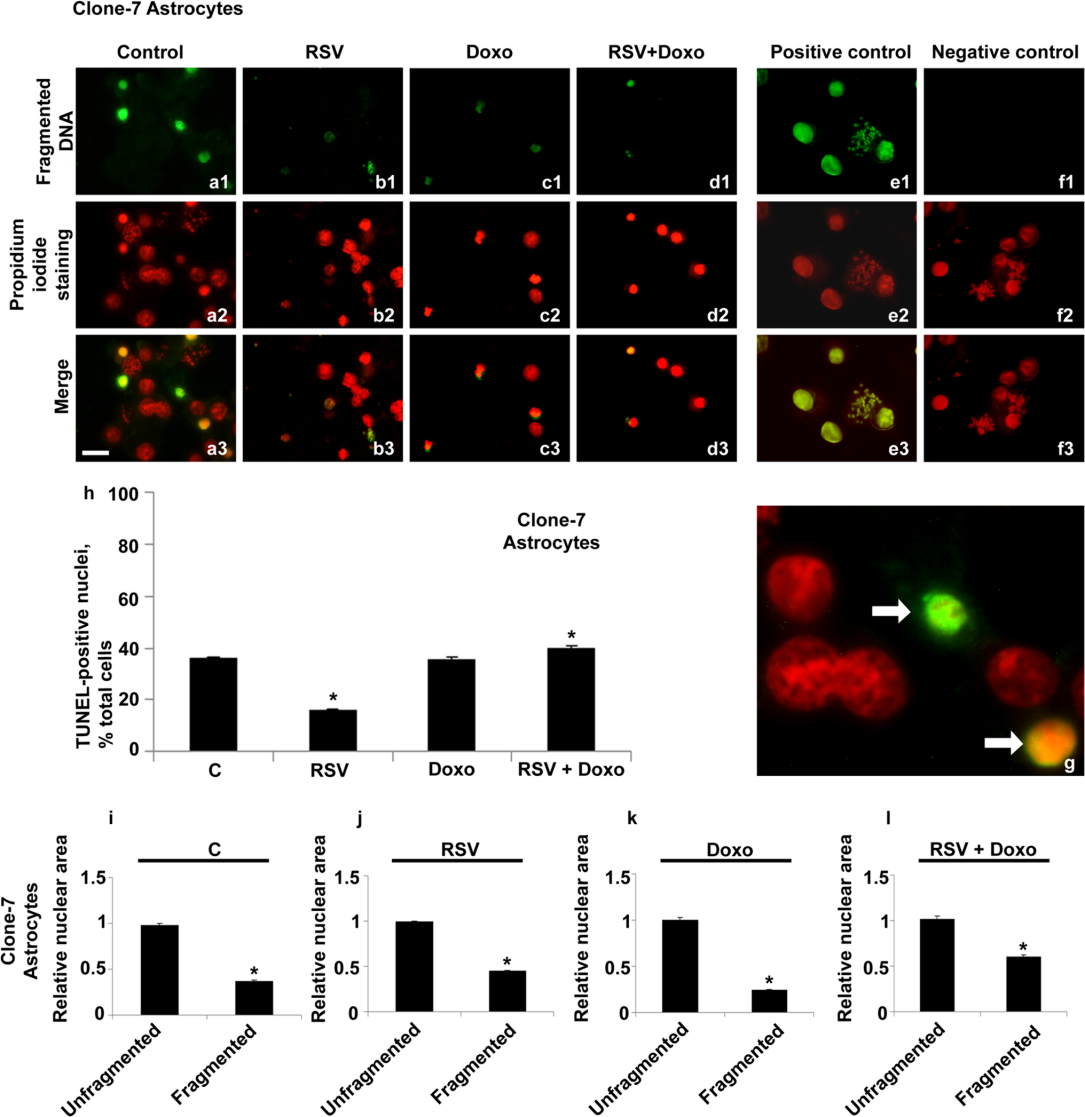
Fluorescent TUNEL assay in astrocytes. Images of representative fields showing nuclei from control and treated cells. DNA fragmentation is shown (**a1**–**f1**). Nuclei marked with propidium iodide (**a2**–**f2**). Merging of both signals (**a3**–**f3** and **g**). Control cells are shown(**a1**–**a3**). RSV-treated cells are shown (**b1**–**b3**). Doxo-treated cells are shown (**c1**–**c3**). Doxo + RSV-treated cells (**d1**–**d3**). The following controls are shown: positive control (**e1**–**e3**), negative control (**f1**–**f3**). Enlargement (**g**) of a particular of control preparation (**a3**). White arrows indicate two fragmented pyknotic nuclei. Scale bar, 20 μm. Histogram showing TUNEL-positive nuclei, percentage of the total cells (**h**). Histograms showing the relative area in unfragmented or fragmented nuclei of control (**i**) and RSV-treated cells (**j**). Doxo-treated cells (**k**). Doxo + RSV-treated cells (**l**). Experiments were performed in triplicate and data are expressed as means ± standard deviation (*n* = 3 ± SD); there were 100 analyzed cells in each experimental group. Asterisks indicate responses significantly different from those of the controls (**h**) and significantly different in fragmented versus unfragmented samples (**i**–**l**) (*p < 0.05)

Chromosomal suspensions and nuclei of clone-7 astrocytes, isolated in our laboratory, were subjected to the TUNEL assay (Fig. 1a–g). In this cell line, DNA fragmentation was only found at the nuclear level, while no metaphase chromosomes with fragmentation points were observed.

An aliquot of 36% of fragmented nuclei was observed in control cells (Fig. 1h).

This percentage decreased to 16% in RSV-treated cells, and it was established to 36% after Doxo treatment. RSV + Doxo cotreatments slightly increased the value to 39%.

In this cell line, a peculiarity about the area of fragmented nuclei was observed. In both the controls and all the treated cells, DNA fragmentation concerned only small-sized nuclei (pyknosis-like nuclei), while all other normal sized nuclei showed unfragmented DNA (Fig. 1g).

The differences between an unfragmented nuclei area and fragmented nuclei area were found significant in all the samples.

The above qualitative and quantitative analyses suggest that in these cells, DNA fragmentation could be related to apoptotic phenomena since the DNA fragmentation signal did not concern the replicating cells (no metaphase chromosomes showed fragmentation points) and all nuclei with characteristics of pyknosis showed fragmented DNA.

**Fig. 2:**
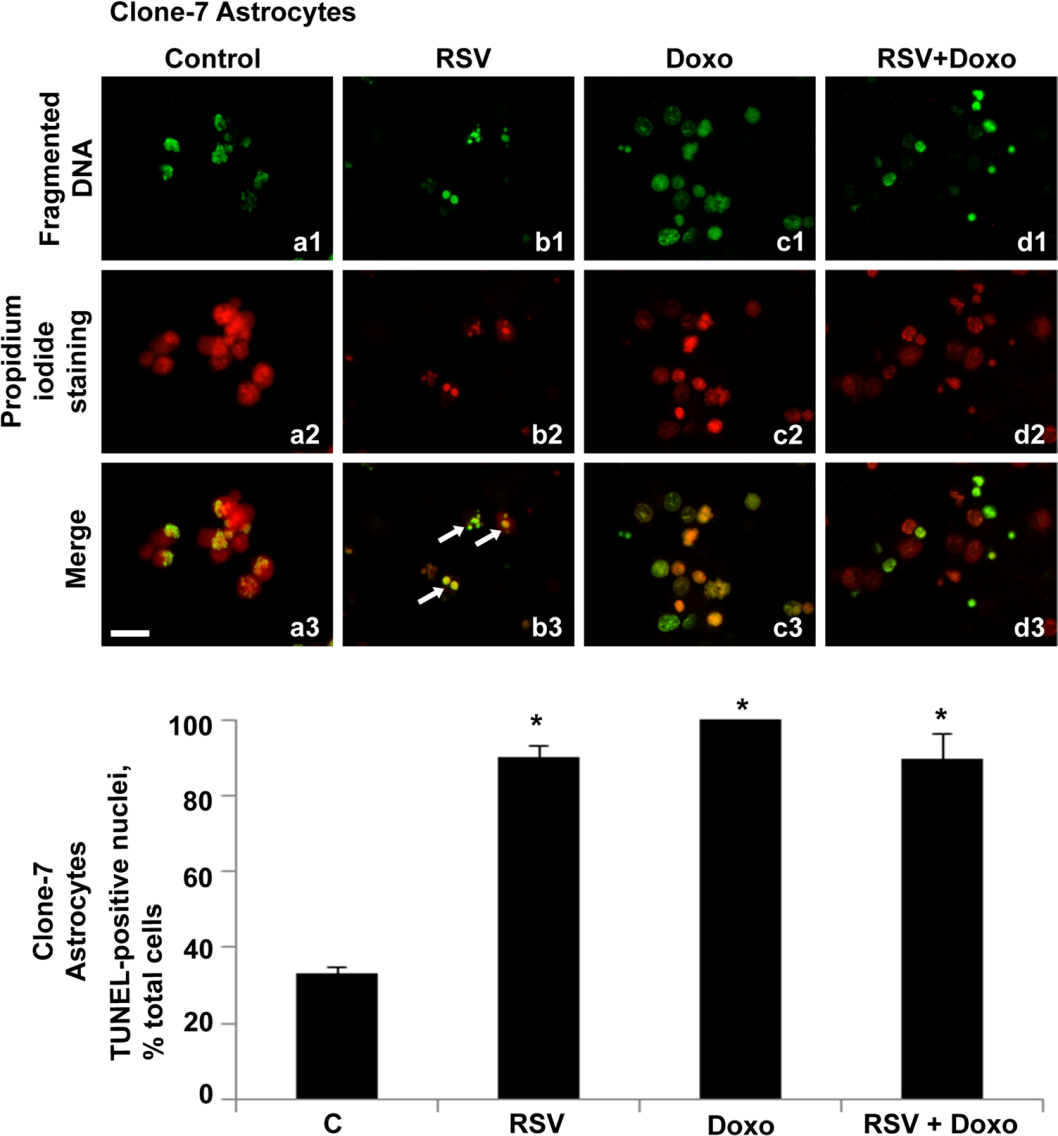
Fluorescent TUNEL assay of detached cellular structures, recovered from astrocytes culture medium. Images of representative fields showing fragmented DNA in control and treated cells. DNA fragmentation (**a1**–**d1**). DNA marked with propidium iodide (**a2**–**d2**). Merging of both signals (**a3**–**d3**). Control samples are shown(**a1**–**a3**). RSV-treated samples (**b1**–**b3**). Doxo-treated samples (**c1**–**c3**). Doxo + RSV-treated samples (**d1**–**d3**). White arrows indicate spherical corpuscles containing fragmented DNA. Scale bar, 20 μm. Histogram showing TUNEL-positive nuclei, percentage of total cells. Experiments were performed in triplicate and data are expressed as mean ± standard deviation (*n* = 3 ± SD); there are 100 analyzed cells in each experimental group . Asterisks indicate responses that are significantly different from those of the controls (*p < 0.05)

Qualitatively, we observed that the culture medium was rich in suspended cellular structures containing DNA. These showed partially fragmented nuclei, as in control cells (Fig. 2a1–a3) and in RSV + Doxo-treated cells (Fig. 2d1–d3). DNA fragmentation and partitioning into small spherical structures were found in RSV-treated cells (Fig. 2b1–b3) and Doxo-treated cells (Fig. 2c1–c3).

For this detached population, we detected high fragmentation rates, about 33% in controls, 90% in RSV-treated cells, 100% in Doxo-treated cells and 89% in RSV + Doxo-treated cells. However, in all treatments, the amount of detached cellular structures with fragmented DNA was always greater and with a statistically significant difference compared with control cells.

### Analysis of DNA fragmentation in Caco-2 cells

A completely different scenario was observed in Caco-2 cells. A peculiar feature was that DNA fragmentation affected chromosomes, uncondensed chromatin nuclei, and, only in treated cells, detached cellular structures.

**Fig. 3:**
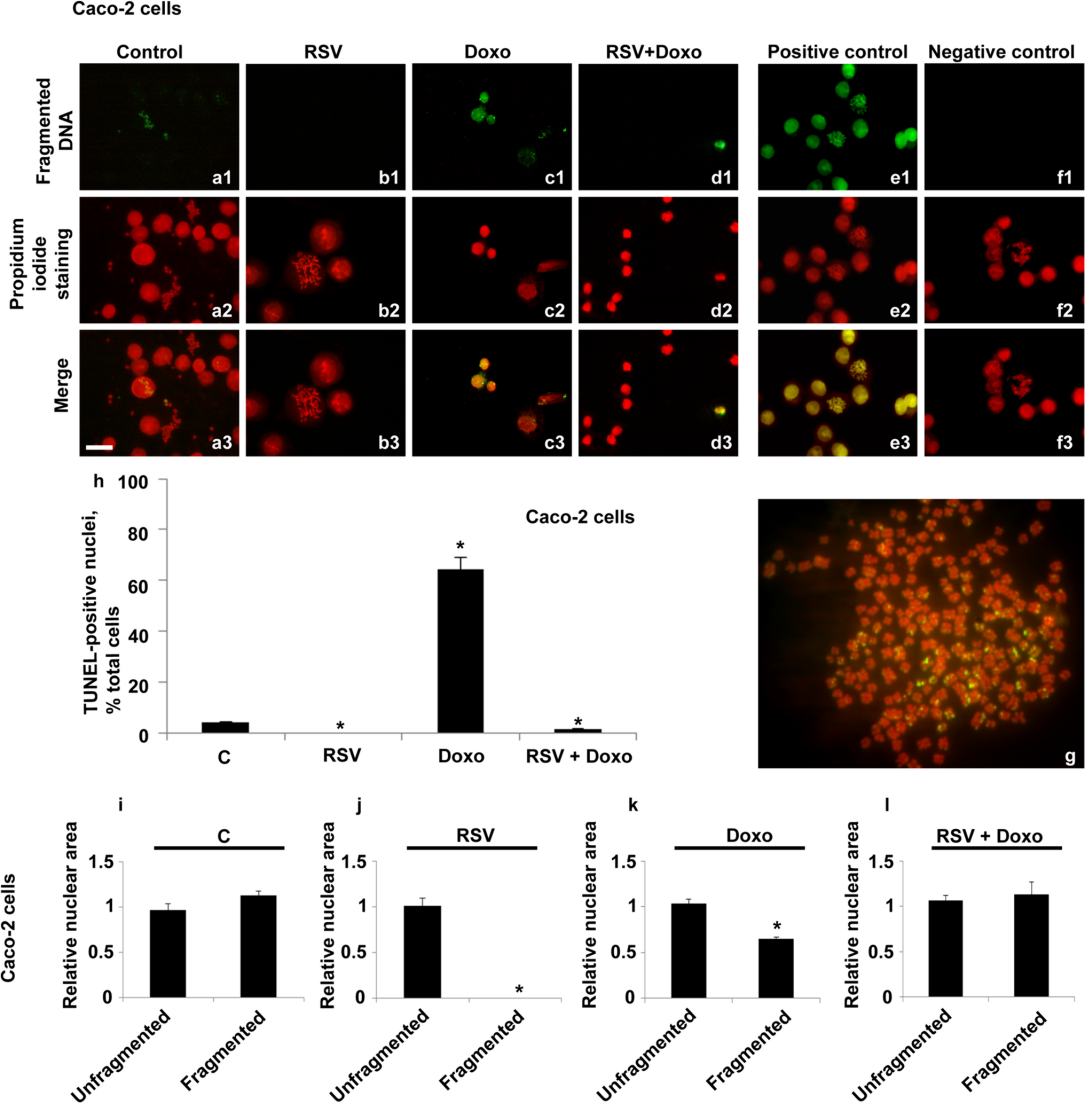
Fluorescent TUNEL assay in Caco-2 cells. Images of representative fields showing nuclei and metaphases from control and treated cells. DNA fragmentation is shown (**a1**–**f1**). DNA marked with propidium iodide (**a2**–**f2**). Merging of both signals (**a3**–**f3** and **g**). Control cells are shown (**a1**–**a3**). RSV-treated cells (**b1**–**b3**). Doxo-treated cells (**c1**–**c3**). Doxo + RSV-treated cells (**d1**–**d3**). Positive control (**e1**–**e3**) and negative control (**f1**–**f3**) are shown. Enlargement of a representative fragmented metaphase of control cells (**g**). Scale bar, 20 μm. Histogram showing TUNEL-positive nuclei, percentage of total cells (**h**). Histograms showing the relative area in unfragmented or fragmented nuclei of control (**i**); RSV-treated cells (**j**). Doxo-treated cells (**k**). Doxo + RSV-treated cells (**l**). Experiments were performed in triplicate and data are expressed as mean ± standard deviation (*n* = 3 ± SD). There are 100 analyzed cells in each experimental group. Asterisks indicate responses significantly different from those of the controls (**h**), and which are significantly different in fragmented versus fragmented samples (**i**–**l**) (*p < 0.05)

In control cells, we observed several metaphases rich in fragmented chromosomes and nuclei with uncondensed chromatin but reporting defined points of fragmentation (Fig. 3a1–a3 and g).

In RSV-treated cells, on the other hand, no DNA fragmentation signals were found in cells adherent to the substrate (Figs. 3b1–b3). In Doxo-treated cells, many nuclei containing fragmented DNA were found with no distinction between normal or pyknotic nuclei. Specifically, the nuclear fragmentation did not concern the whole area but only some points that appeared as fragmented spots (Figs. 3c1–c3). Cells exposed to the combined treatment (RSV + Doxo), however, showed nuclei with more diffuse fragmented DNA (Figs. 3d1–d3).

As reported in the histogram (Fig. 3h), a high percentage of nuclei containing fragmented DNA (66%) was found only in Doxo-treated cells, for which, compared to control, a statistically significant difference was found.

Analyzing the area of fragmented and unfragmented nuclei, no statistically significant difference was found between the two parameters (Figs. 3i–l), except for Doxo-treated cells and excluding RSV-treated cells, which had no nuclei containing fragmented DNA. Consequently, since metaphase chromosomes represent a clear sign of cell replication, the presence of DNA fragmentation, also in chromosomes, suggested an unlikely relationship with the ongoing death phenomena.

**Fig. 4:**
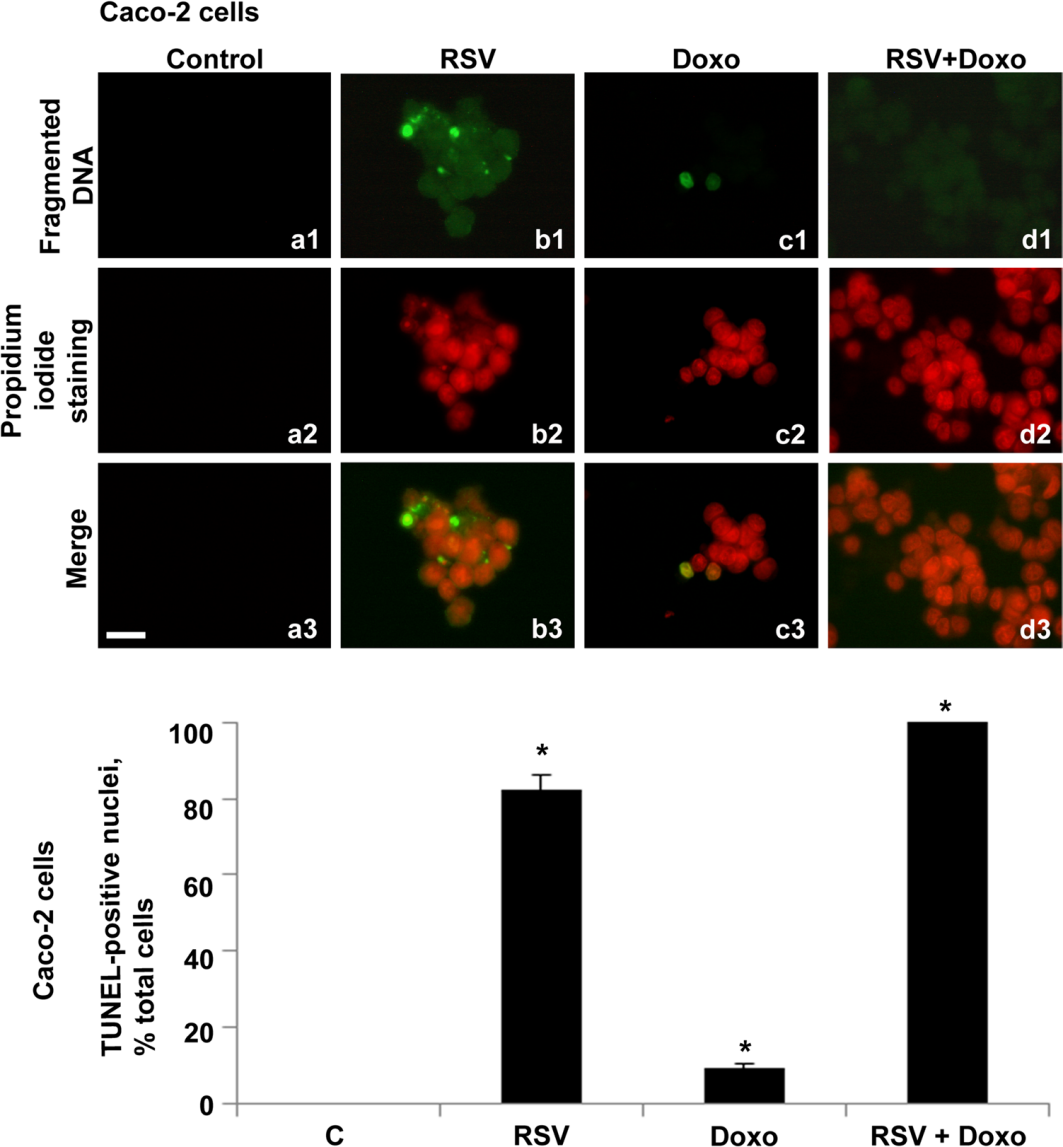
Fluorescent TUNEL assay in detached cellular structures, recovered from Caco-2 cells’ culture medium. Images of representative fields showing fragmented DNA in control and treated cells. DNA fragmentation is shown (**a1**–**d1**). DNA stained with propidium iodide (**a2**–**d2**). Merging of both signals (**a3**–**d3**). Control samples are shown (**a1**–**a3**). RSV-treated samples (**b1**–**b3**). Doxo-treated samples (**c1**–**c3**). Doxo + RSV-treated samples (**d1**–**d3**). Scale bar, 20 μm. Histogram showing TUNEL-positive nuclei, percentage of total cells. Experiments were performed in triplicate and data are expressed as mean ± standard deviation (*n* = 3 ± SD). There are 100 analyzed cells in each experimental group. Asterisks indicate responses that are significantly different from those of the controls (*p < 0.05)

In parallel, an analysis on detached cellular structures was performed. No fragmented or unfragmented nuclear structures were detected in the medium from control cells (Figs. 4a1–a3). Large clusters of nuclei with a high number of fragmented DNA spots were found in the medium of RSV-treated cells (Figs. 4b1–b3). Groups of nuclei with a lower fragmentation rate than the RSV-treated ones were identified in the culture medium recovered from Doxo-treated cells (Figs. 4c1–c3). A high number of nuclei was found in the culture medium of cells treated with RSV + Doxo. All these nuclei exhibited diffuse fragmentation, albeit with a very slight intensity (Figs. 4d1–d3).

### Analysis of DNA Fragmentation in MDA–MB–231 cells

**Fig. 5:**
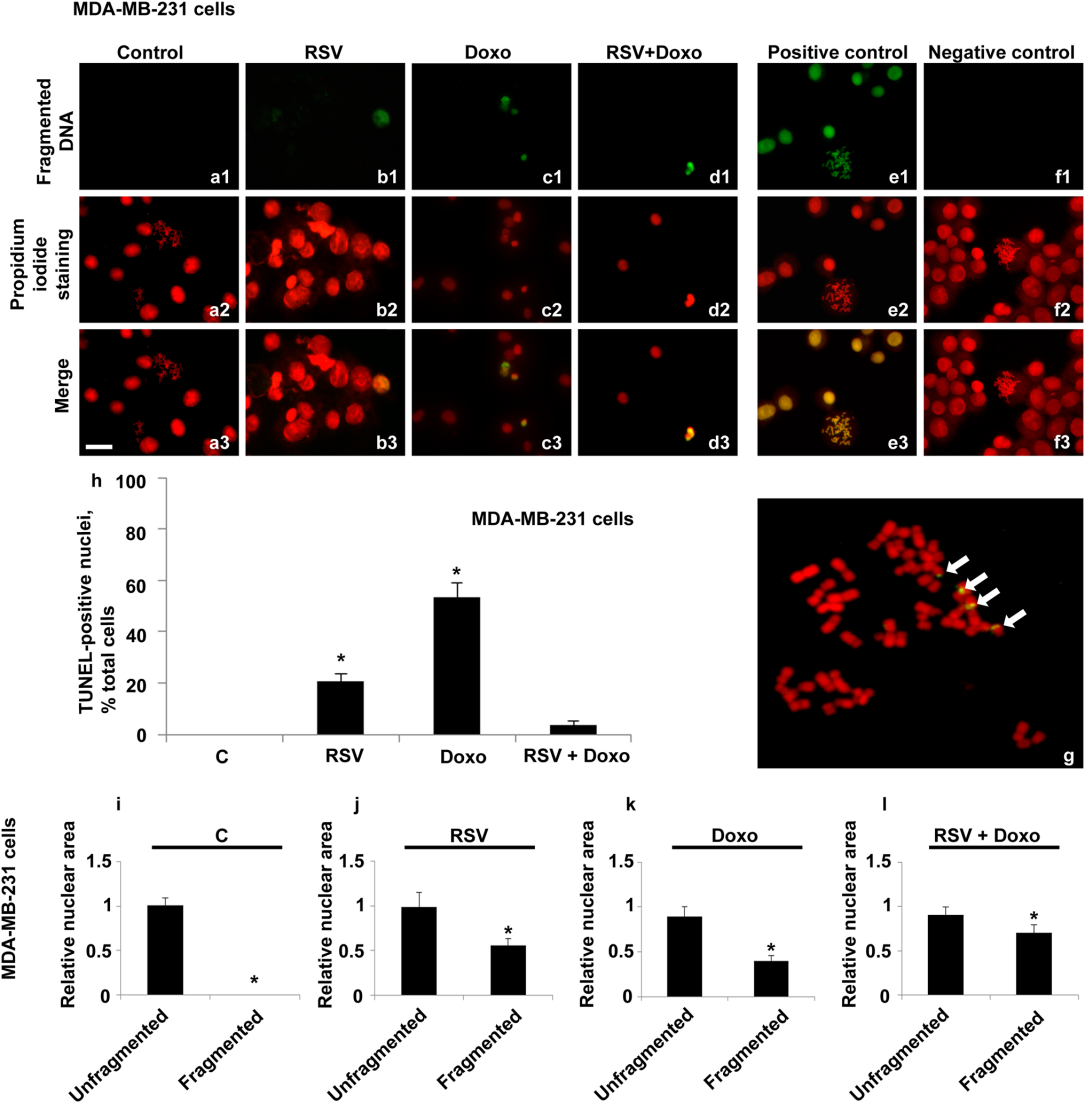
Fluorescent TUNEL assay in MDA-MB-231 cells. Images of representative fields showing nuclei and metaphases from control and treated cells. DNA fragmentation is shown (**a1**–**f1**). DNA stained with propidium iodide (**a2**–**f2**). Merging of both signals (**a3**–**f3** and **g**). Control cells are shown(**a1**–**a3**). RSV-treated cells (**b1**–**b3**). Doxo-treated cells (**c1**–**c3**). Doxo + RSV-treated cells (**d1**–**d3**). Positive controls (**e1**–**e3**) and negative controls (**f1**–**f3**) are shown. Enlargements of a representative fragmented metaphase of control cells (**g**). White arrows indicate chromosomes with fragmented points. Scale bar, 20 μm. Histogram showing the percentage of fragmented nuclei (**h**). Histogram showing TUNEL-positive nuclei, percentage of total cells (**i**); RSV-treated cells (**j**). Doxo-treated cells (**k**). Doxo + RSV-treated cells (**l**). Experiments were performed in triplicate and data are expressed as mean ± standard deviation (*n* = 3 ± SD). There are 100 analyzed cells in each experimental group. Asterisks indicate responses significantly different from those of the controls (h) and that are significantly different in fragmented versus unfragmented samples (**i**–**l**) (*p < 0.05)

Considering the DNA damage, MDA–MB–231 cells showed a different behavior compared with the other two cell lines. The control cells did not show signals of DNA fragmentation, neither at the nuclei level nor at the chromosomes level (Fig. 5a1–a3). Following treatment with RSV, many nuclei containing fragmented DNA were observed (Fig. 5b1–b3). The nuclei fragmentation rate was increased following treatment with Doxo (Fig. 5c1–c3) and greatly reduced in RSV + Doxo cotreated cells. Surprisingly, only 1% of the analyzed metaphases showed some fragmented chromosomes (Fig. 5g). The increase in nuclear fragmentation reached statistical significance, especially for cells exposed to RSV or Doxo (Fig. 5h).

A relationship was observed between the reduction of the nuclei area and the DNA fragmentation. As reported in the histograms (Fig. 5i–l), the differences between unfragmented nuclei area and fragmented nuclei areas in all the samples were found to be significant.

These data suggest that in MDA–MB–231 cells, DNA fragmentation could be related to apoptotic phenomena, although some cells showed fragmented chromosomes, indicative of DNA damage in replicating cells.

**Fig. 6:**
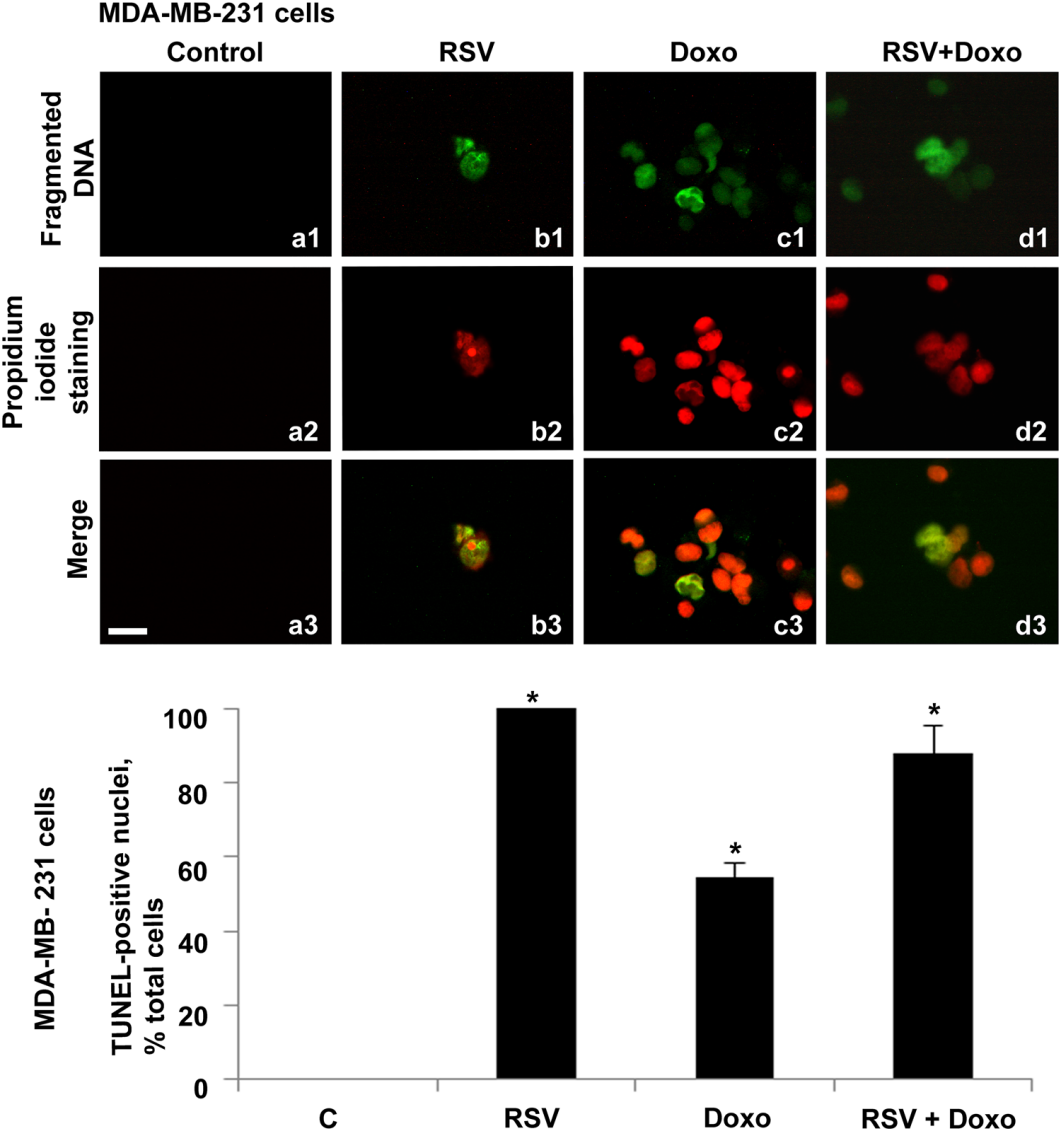
Fluorescent TUNEL assay of detached cellular structures, recovered from MDA-MB-231 medium culture. Images of representative fields showing fragmented DNA from control and treated cells. DNA fragmentation is shown (**a1**–**d1**). DNA stained with propidium iodide (**a2**–**d2**). Merging of both signals (**a3**–**d3**). Control samples are shown (**a1**–**a3**). RSV-treated samples (**b1**–**b3**). Doxo-treated samples (**c1**–**c3**). Doxo + RSV-treated samples (**d1**–**d3**). Scale bar, 20 μm. Histogram showing TUNEL-positive nuclei, percentage of total cells. Experiments were performed in triplicate and data are expressed as mean ± standard deviation (*n* = 3 ± SD). There are 100 analyzed cells in each experimental group. Asterisks indicate responses that are significantly different from those of the controls (*p < 0.05)

Except for control cells, numerous cellular structures (apoptotic body-like corpuscles) were observed in all treatments in the suspended cell population belonging to the MDA–MB–231 cell line (Fig. 6).

As shown in the histogram, 100% of fragmented spots for RSV-treated cells, 58% for Doxo-treated cells, and 88% for combined RSV + Doxo-treated cells was observed.

Since the culture medium recovered from RSV-treated cells showed a greater variety of detached cellular structures, a more detailed analysis was performed.

**Fig. 7:**
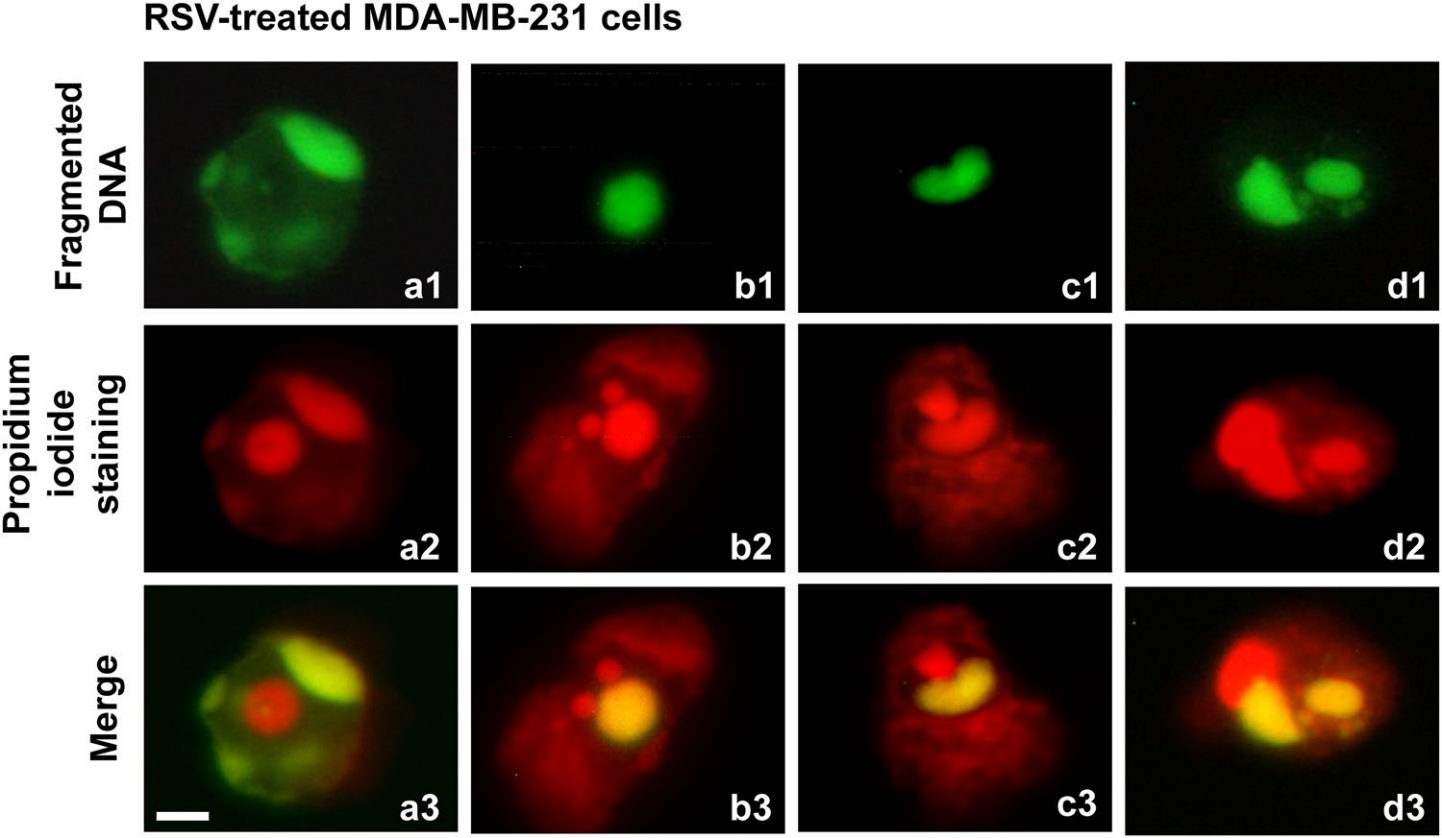
Fluorescent TUNEL assay of detached cellular structures, recovered from MDA-MB-231 cells’ culture medium. Images of representative fields showing fragmented DNA from RSV-treated cells. DNA fragmentation is shown (**a1**–**d1**). DNA stained with propidium iodide (**a2**–**d2**). Merging of both signals (**a3**–**d3**). Nuclear structure with unfragmented central body and fragmented peripheral DNA islands (**a1**–**a3**). Nuclear structure with fragmented central body and two agglomerates of unfragmented peripheral DNA (**b1**–**b3**). Nuclear structure divided into two compartments of fragmented and unfragmented DNA (**c1**–**c3**). Nuclear structure was divided into three compartments, one with unfragmented DNA and two with fragmented DNA (**d1**–**d3**). Scale bar, 5 μm

In all cases, a breakdown of fragmented DNA within the nuclear structure was observed, highlighting the simultaneous presence of intact DNA and fragmented DNA. The latter appeared in spherical body structures clearly detached from the portions of unfragmented DNA (Figs. 7a1–d3).

By considering these morphological aspects, it seems that this cell line is very active in eliminating cells with fragmented DNA (which we found in detached cellular structures), but at the same time, although in a small percentage of the replicating cells showed fragmented DNA as chromosomal evidence.

## Discussion

The characterization of cell line markers is of considerable importance in biomedical research (Geraghty et al. 2014). This aspect arouses considerable interest when the toxic or beneficial effects induced by new chemical agents need to be tested. Moreover, it is a common notion that different cell lines respond differently to cytotoxic stimuli.

Numerous experimental approaches show that the genetic characterization of cells, as well as the karyotype analysis, needs to be coupled to other markers (Gäberlein et al. 2023).

DNA is the only molecule in the cell that can be repaired but cannot be completely resynthesized after damage (Moore et al. 2021).

Most of the experimental investigations in the literature aim to observe variations in gene expression or in the modulation of proteins marker levels. The presence of genome fragmentation points represents a difficult component to study since there are no unique and indicative markers of this damage.

DNA damage is one of the main markers that can be used to study multiple effects induced by chemical or physical agents in a multifactorial and extremely complex process. The multifactoriality derives from the fact that DNA fragmentation can be triggered following an exogenous factor, but also in the context of programmed cell death phenomena. In fact, final stages of apoptosis involve the DNA fragmentation and the structuring of chromatin inside apoptotic bodies, particularly extracellular vesicles that could be involved in both programmed cell death and also intercellular communication (Battistelli and Falcieri 2020).

Apoptosis is an active process that occurs in normal cell turnover as well as in stress-induced cell death, representing a homeostatic mechanism for maintaining regular cell populations in tissues (Atkin-Smith and Poon 2017).

Apoptosis progresses through several stages, the first being nuclear chromatin condensation, then nuclear splitting and the frequent appearance of micronuclei and corpuscles containing chromatin remnants, cytosol portions, degraded proteins and DNA fragments.

DNA fragmentation can affect replicating cells and, in this case, the damage can be repaired or transmitted to daughter cells (Huang and Zhou 2021).

Another important point is the research of fragmented DNA in detached cells, since this data contribute to the definition of global toxicity induced by chemicals. In fact, the conditioned medium is a reservoir of useful information that until now was excluded from the various analyses, as reported by Rosochowicz et al. (2023).

In the past, we determined genetic details about three rat astrocyte cell lines with increasingly transformed properties and analyzed them at both cytogenetic, epigenetic (Caradonna et al. 2020), and proteomic levels (Schiera et al. 2023). We found that the most modified cell line (A-FC6) showed epigenetic and chromosomal alterations typical of tumor cells, such as the cytogenetic marker i(8q) in 100% of metaphases. Subsequently, we isolated the clone-7 astrocytes selected for a shortening of cell doubling time. This clone further showed the same epigenetics features of A-FC6 and more,interestingly, the cytogenetic marker i(8q) was present in double copy in about 15% of metaphases (data not shown). In a schematic line of increasing genomic instability, we put the clone-7 astrocytes at the second place, after the normal cells. Therefore, regarding the here described in vitro model system, starting from the normality to the genetically more unstable transformed cells, we assign the point “0” to normal cells and the point “1” to the clone-7 astrocytes.

It is known the peculiarity of the widely used Caco-2 cell line. These cells, although tumoral, assume a different behavior in long-term cultures, as they show the ability to differentiate into “normal” cells. It is evident that their genomic instability is still at reversible levels, far from that of an aggressive tumor cell that does not possess this possibility. For this peculiarity we assign to these cells, here cultured for the short-term, the point “2” of the described in vitro model system.

The MDA-MB-231 triple negative breast cancer cell line is composed of cells with a high degree of genomic instability and also a high metastatic potency (Huang et al. 2020). For these features, we assign to this cell line the point “3” of the described in vitro model system.

In the present paper, the comparative analysis carried out with three different cell lines (clone-7 astrocytes, Caco-2 cells, and MDA-MB-231 cells) proposes a model of increasing genomic instability. We report that cells respond in a different way to the effects induced by toxic agents, since there were different levels of fragmentation in nuclear DNA (whole nuclei), single chromosomes, and DNA associated with extracellular structures (recovered from the culture medium). Although our aim was not to study apoptosis but the presence of DNA damage at different levels, in some cases we noticed that such apoptotic body-like structures were abundantly present in the culture medium and were of considerable importance for the overall study of DNA fragmentation.

Our integrated and comparative approach allows us to highlight some key points. In many cases, when apoptotic phenomena or other DNA fragmentation processes are investigated, it is not enough to study the phenomena at the cellular level, but it is necessary to integrate the data with the phenomena that are occurring at the extracellular level.

As an example, clone-7 astrocytes showed a nuclear fragmentation rate in adherent cells, both in controls and in Doxo-treated and RSV + Doxo cotreated cells ranging from 16% to 40%. Integrating the data obtained from the culture medium, compared with the control, we note that all the treated cells showed a nuclear fragmentation rate equal to about 1.5 times higher than those treated with RSV and about 2 times higher than those treated with Doxo and cotreated with RSV + Doxo. These cells, overall, were more sensitive to the exposure of Doxo.

On the other hand, Caco-2 cells showed a low rate of nuclear fragmentation in substrate-adherent cells, except for Doxo-treated cells, for which a value equal to about 65% was reached. Integrating the data detected by the analysis of the culture medium, we noted that in the controls we did not detect any signal of DNA fragmentation (this is in accordance with the type of fragmentation, which involved replicating cells and not dying cells, given that the points of fragmentation were evident in metaphase chromosomes). Although RSV− and RSV+ Doxo-treated cells did not show a high level of nuclear fragmentation in substrate-attached cells, with our integrated analysis we noted that in the culture medium the rate of nuclei containing fragmented DNA reached values ranging from 20 to 25-fold higher than those of the control, proving that the nuclear fragmentation phenomena were in an advanced stage, such as to promote a loss of adhesion of the cells. The cells exhibited sensitivity to both RSV and Doxo, to such an extent that cotreatment of RSV + Doxo resulted in increased nuclear fragmentation.

The MDA–MB-231 control cells did not exhibit nuclear fragmentation in the substrate-adherent cells. These cells showed a high rate of nuclear fragmentation after exposure to Doxo (about three times higher compared with those treated with RSV alone). In cotreatment, at least for substrate-adherent cells, the nuclear fragmentation rate was basal. By integrating the data obtained from the culture medium, we noted that there was a compensation as the levels of nuclear fragmentation were low in the cells that showed high values of nuclear fragmentation at the level of substrate-adherent cells (Doxo-treated cells) and high in cells showing low levels of nuclear fragmentation related to substrate-adherent cells (RSV-treated and RSV + Doxo cotreated cells). This integrated view allows us to understand that these cells are sensitive to treatment with both molecules, with a slightly higher entity for treatments with RSV.

## Conclusions

The data, obtained with a modern revisitation of a TUNEL assay, showed a comparative vision of three genomically different cell lines and allowed us to gain data for the study of DNA fragmentation at different levels. Through our analysis, we were able to observe that the clone-7 astrocytes were the only cells demonstrating the absence of DNA fragmentation at the chromosomal level. These cells presented chromatin condensation and pyknotic nuclei corresponding to the fragmented DNA signals. This aspect appeared to be present in control cells and was modulated by exposure to single or combined treatments with RSV/Doxo. No chromosomal fragmentation was found in any case. Evidently, the cells choose to either repair the damage or simply activate apoptotic phenomena. Caco-2 cells showed fragmented metaphase chromosomes, proving that the DNA damage was transmitted to the daughter cells. The treatments seemed to reduce this effect and, in particular, RSV promoted a major induction of fragmentation at the nuclear level, most noticeable in the suspended than in the substrate-adherent cells. Thus, we can hypothesize that Caco-2 cells have no DNA repair mechanism activated. However, MDA–MB-231 cells, analyzed by this alternative TUNEL assay approach, showed a very low or no fragmented metaphases, suggesting a probable activation of DNA repair mechanisms.

Overall from all these results we may assert that the three cell lines respond differently in terms of DNA fragmentation, after RSV and DOXO treatments and the RSV–DOXO cotreatment, probably based on their biological status.

By applying, for the first time, the well-known TUNEL test on nuclei and metaphases, we obtained data relating to DNA fragmentation that can significantly contribute to characterizing the tumor phenotype of a cell type, either in a clinical setting or to test the effect of candidate molecules such as new anticancer drugs.

## Data Availability

The data and material supporting the findings of this study are available from the corresponding author, Fabio Caradonna, upon request.

## Abbreviations

TUNEL: Terminal deoxynucleotidyl transferase dUTP nick end labeling
RSV: Resveratrol
Doxo: Doxorubicin
GI: Genomic instability
NGS: Next-generation sequencing
DMEM: Dulbecco’s modified eagle’s medium
DMSO: Dimethyl sulfoxide
IC50: Half maximal inhibitory concentration
PBS: Phosphate-buffered saline
rTdT: Enzyme terminal deoxynucleotidyl transferase, recombinant
SSC: Saline-sodium citrate
DABCO: 1,4-DiAzaBicyclo ( 2–2-2) octane
SD: Standard deviation
